# From treating pain to promoting health: a competency-based salutogenic framework for pain medicine education

**DOI:** 10.1093/heapro/daag128

**Published:** 2026-08-28

**Authors:** Cecília Daniele de Azevedo Nobre, Bruno Vítor Martins Santiago, Bruno Augusto Parada, Carlos Alves Darcy Bersot, Lara Calainho de Oliveira, João Vítor de Araujo Gonçalves, Liszt Palmeira de Oliveira, Hazem A Ashmawi

**Affiliations:** Department of Physical Medicine and Rehabilitation, State University of Rio de Janeiro (UERJ), Boulevard 28 de Setembro, 77, Vila Isabel, Rio de Janeiro, RJ 20551-030, Brazil; Department of Orthopedics and Traumatology, School of Medical Sciences, State University of Rio de Janeiro (UERJ), Boulevard 28 de Setembro, 77, Vila Isabel, Rio de Janeiro, RJ 20551-030, Brazil; Department of Anesthesiology, Pedro Ernesto University Hospital, State University of Rio de Janeiro (UERJ), Boulevard 28 de Setembro, 77, Vila Isabel, Rio de Janeiro, RJ 20551-030, Brazil; Pain Clinic, Pedro Ernesto University Hospital, State University of Rio de Janeiro (UERJ), Boulevard 28 de Setembro, 77, Vila Isabel, Rio de Janeiro, RJ 20551-030, Brazil; Department of Anesthesiology, Federal University of São Paulo (UNIFESP), Rua Botucatu, 740, 5th floor, Room 562, Octávio de Carvalho Building, Vila Clementino, São Paulo, SP 04023-062, Brazil; School of Medical Sciences, State University of Rio de Janeiro (UERJ), Boulevard 28 de Setembro, 77, Vila Isabel, Rio de Janeiro, RJ 20551-030, Brazil; School of Medicine and Surgery, Gaffrée e Guinle University Hospital, Federal University of the State of Rio de Janeiro (UNIRIO), Rua Mariz e Barros, 775, Maracanã, Rio de Janeiro, RJ 20270-901, Brazil; Department of Orthopedics and Traumatology, School of Medical Sciences, State University of Rio de Janeiro (UERJ), Boulevard 28 de Setembro, 77, Vila Isabel, Rio de Janeiro, RJ 20551-030, Brazil; Department of Anesthesiology, University of São Paulo (USP), Avenida Professor Lineu Prestes, 2565, Cidade Universitária, Butantã, São Paulo, SP 05508-000, Brazil

**Keywords:** health promotion, salutogenesis, chronic pain, pain medicine, medical education, competency-based medical education, sense of coherence, resilience, therapeutic alliance

## Abstract

Chronic pain is a leading cause of disability worldwide and remains a challenge for healthcare systems, medical education, and public health. Although advances in pain neuroscience and interventional therapies have improved pain management, biomedical approaches alone are insufficient to address the multidimensional nature of persistent pain or promote adaptive recovery, participation, and long-term functioning. Contemporary pain medicine therefore requires educational models integrating health promotion, resilience, relationship-centered care, and sustainable professional practice. To develop and describe a competency-based salutogenic educational framework for pain medicine integrating contemporary concepts from pain science, health promotion, behavioral science, and medical education. The framework was developed through conceptual integration of complementary theories from pain medicine, psychology, health promotion, and competency-based medical education. It combines total pain, salutogenesis and sense of coherence, resilience, Social Cognitive Theory, therapeutic alliance, and physician professional sustainability within a longitudinal educational structure. Rather than replacing existing biopsychosocial or salutogenic models, it integrates their complementary strengths by explicitly linking adaptive recovery, relationship-centered care, competency development, authentic assessment, and health promotion. Educational implications include longitudinal competency-based curricula, reflective practice, interdisciplinary learning, patient partnership, workplace-based assessment, and validated psychometric instruments alongside competency-based educational assessments to evaluate learner and patient outcomes. The proposed framework reorients pain medicine education beyond symptom management toward adaptive recovery, participation, equity, and person-centered care. By integrating health promotion with competency-based medical education, it provides a conceptual foundation for curriculum development, implementation research, and evaluation of educational strategies to improve outcomes for patients, healthcare professionals, and health systems.

## Graphical Abstract

**Graphical Abstract:**
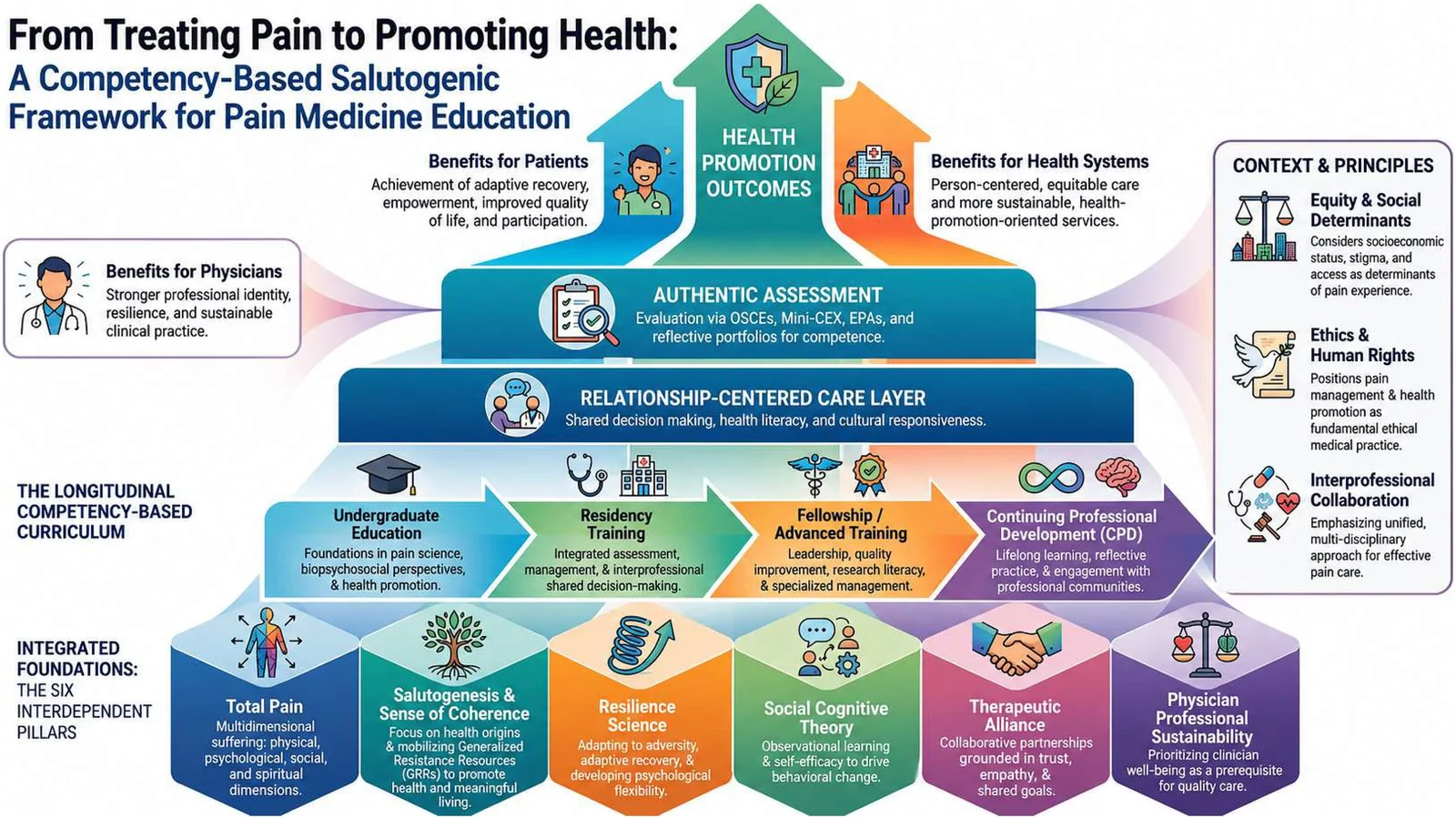

Contribution to Health Promotion

- Proposes an integrated competency-based salutogenic framework that brings together complementary concepts from pain science, health promotion, and medical education.
- Integrates Total Pain, Salutogenesis, resilience science, Social Cognitive Theory, therapeutic alliance, and physician professional sustainability into a “coherent competency-based educational architecture.”
- Supports longitudinal competency-based and relationship-centered educational strategies for chronic pain care.
- Strengthens health promotion by incorporating health equity, social determinants of health, patient partnership, and interdisciplinary collaboration.
- Provides a practical roadmap for curriculum development, “authentic competency-based assessment,” and future implementation research.

## Introduction

Chronic pain is one of the leading causes of disability worldwide and a major challenge for health systems, medical education, and health promotion (Raja *et al*. 2020, Cohen *et al*. 2021, GBD 2021 Diseases and Injuries Collaborators 2024). Affecting nearly one in five adults, persistent pain compromises physical functioning, psychological well-being, social participation, and quality of life. Consequently, it should be understood as a complex health condition rather than merely a symptom, requiring educational approaches that promote adaptive functioning, resilience, participation, and long-term well-being alongside symptom management (World Health Organization 1986, Nutbeam 2000).

Despite substantial advances in pain neuroscience, pharmacology, and interventional therapies, outcomes remain unsatisfactory for many individuals, a phenomenon described as the treatment–prevalence paradox (Johnson 2025a). Although Engel's biopsychosocial model transformed pain medicine by integrating biological, psychological, and social determinants (Engel 1977), its implementation in education has often remained fragmented. Increasingly, contemporary pain science has evolved toward salutogenic and health-promoting perspectives that emphasize adaptive recovery, resilience, person-centered care, and participation as essential components of long-term pain management (Antonovsky 1979, Mittelmark and Bauer 2017, Johnson 2025a). This evolution reflects a broader shift from disease-oriented education toward educational models that explicitly promote health and adaptive functioning.

This conceptual evolution has generated complementary frameworks that have expanded understanding of persistent pain from different theoretical perspectives. PRISM emphasizes adaptive recovery through resilience-oriented education (Tatta *et al*. 2023); Johnson's integral perspective situates pain within a salutogenic social model of health (Johnson 2025a, 2025b); and the renewed interpretation of Total Pain extends multidimensional suffering beyond palliative care to chronic pain (Saunders 1963, Goebel *et al*. 2009, Rattner 2023, Habib *et al*. 2026). Although these models have substantially advanced contemporary pain science, they represent complementary conceptual perspectives rather than integrated educational frameworks for competency-based pain medicine.

However, these perspectives have largely evolved in parallel. While each contributes important theoretical insights, no existing framework explicitly integrates their complementary educational contributions within a competency-based architecture for pain medicine that combines Total Pain, Salutogenesis and Sense of Coherence, resilience science, Social Cognitive Theory, therapeutic alliance, physician professional sustainability, and longitudinal competency development. Rather than viewing these concepts as competing paradigms, they may be understood as complementary domains that address different dimensions of learning, clinical practice, and professional development. Consequently, educational programs continue to prioritize biomedical knowledge and procedural competence while giving comparatively less attention to adaptive recovery, therapeutic alliance, patient empowerment, shared decision-making, health literacy, resilience, and the social determinants of health.

This Perspective addresses this educational gap by proposing a competency-based salutogenic framework that integrates these complementary conceptual traditions within an explicit health promotion perspective. Rather than introducing a new theory of pain or replacing existing conceptual models, the proposed framework provides a coherent educational architecture intended to support longitudinal curriculum development, educational implementation, competency-based assessment, and future implementation research in pain medicine education. By positioning health promotion as an educational principle rather than an additional curricular component, the framework seeks to strengthen the translation of contemporary pain science into educational practice.

Conceptual frameworks require transparent justification regarding the selection and integration of their theoretical foundations. Accordingly, the present framework was developed through an iterative narrative synthesis of complementary literature from pain medicine, health promotion, behavioral science, and medical education, with the explicit aim of identifying educational domains capable of supporting competency-based pain medicine education. Because the objective was conceptual integration rather than evidence synthesis, a narrative approach was considered the most appropriate methodology for framework development.

Framework development was guided by three predefined criteria: conceptual complementarity, educational applicability, and clinical relevance. Conceptual complementarity referred to the ability of individual theories to explain distinct yet interconnected dimensions of persistent pain and professional development. Educational applicability considered whether theoretical constructs could be translated into competencies, teaching strategies, curriculum design, and learner assessment. Clinical relevance reflected consistency with contemporary person-centered, biopsychosocial, and health-promoting approaches to persistent pain. Together, these criteria were intended to ensure that the selected domains contributed complementary educational functions rather than overlapping theoretical perspectives.

Based on these criteria, six complementary conceptual domains were selected: Total Pain, Salutogenesis and Sense of Coherence, resilience science, Social Cognitive Theory, therapeutic alliance, and physician professional sustainability. These domains were selected because each addresses a distinct dimension of competency development, collectively encompassing multidimensional pain understanding, adaptive health resources, behavioral learning, relationship-centered care, and sustainable professional practice.

The framework was refined through iterative comparison of the theoretical assumptions, educational implications, and clinical applications of these domains. Particular attention was given to areas of conceptual convergence, including adaptive recovery, patient agency, meaning-making, resilience, therapeutic relationships, and lifelong professional development. These shared principles were synthesized into a competency-based educational framework designed to support longitudinal competency development rather than isolated theoretical instruction. The resulting framework should therefore be interpreted as a conceptual educational architecture that generates hypotheses for curriculum development and implementation research, rather than as a validated educational model.

**Figure 1:**
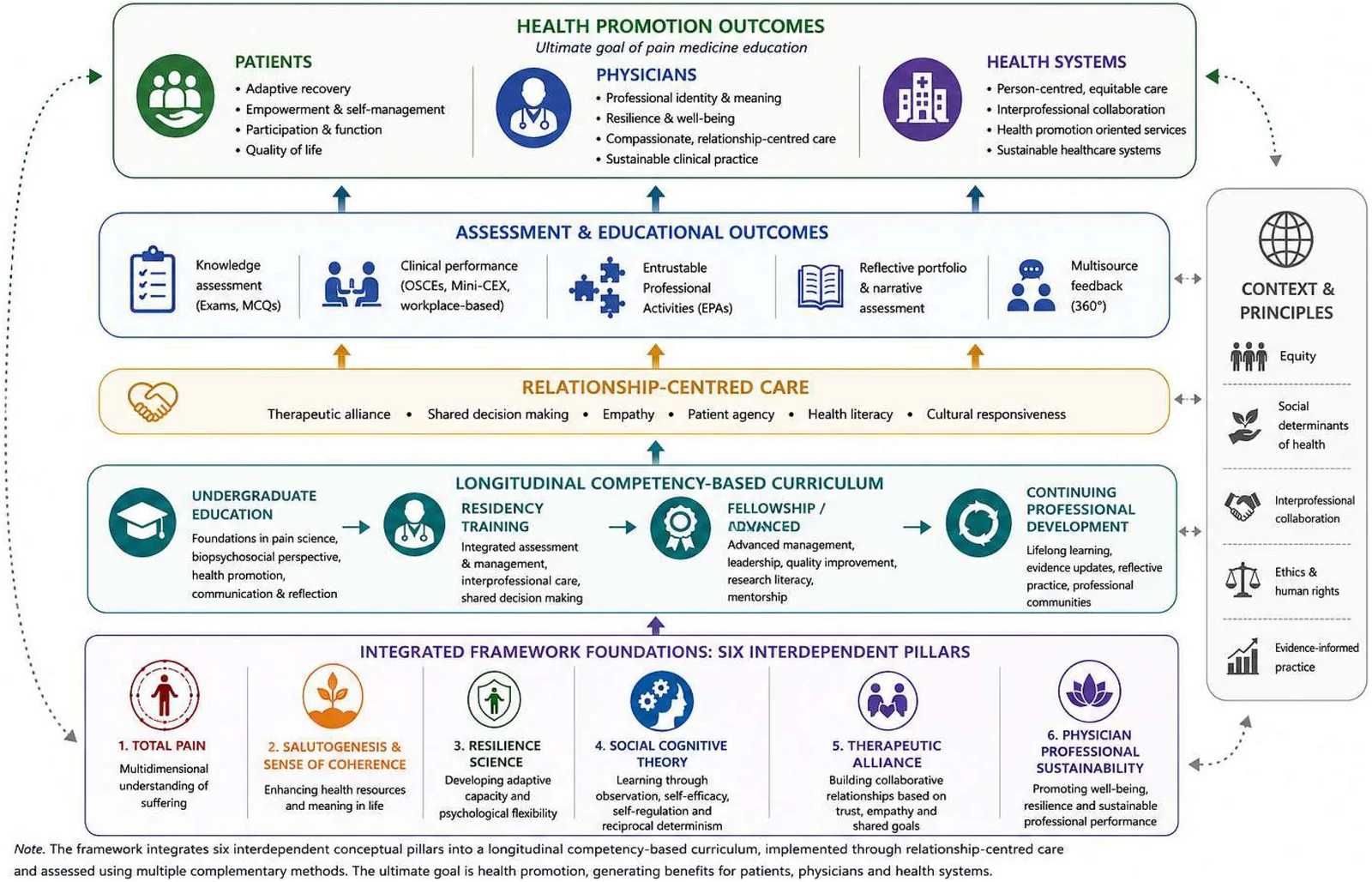
Proposed integrated health-promoting framework for competency-based pain medicine education. The model integrates six complementary conceptual pillars into a longitudinal competency-based curriculum supported by relationship-centered care, educational strategies, and authentic assessment, with the aim of promoting adaptive recovery, patient empowerment, physician sustainability, and health-promoting healthcare systems. OSCE, Objective Structured Clinical Examination; EPA, Entrustable Professional Activity.

Figure 1 illustrates the proposed conceptual architecture. Rather than depicting independent conceptual models, it illustrates how the six complementary domains interact within a longitudinal competency-based curriculum supported by learner-centered educational strategies, authentic workplace-based assessment, and relationship-centered care. “The directional relationships represented in the figure are conceptual and hypothesis-generating, rather than empirically validated causal pathways.” Health promotion functions as the overarching educational objective, linking competency development with adaptive recovery, patient empowerment, physician well-being, and sustainable healthcare systems.

## The evolution of health-promoting pain education

Over the past five decades, pain medicine has progressively evolved from a predominantly biomedical discipline toward more comprehensive, person-centered models of care. Engel's biopsychosocial model represented a major conceptual advance by recognizing that biological, psychological, and social factors interact dynamically in shaping the pain experience (Engel 1977). Although this model transformed clinical reasoning, its educational implementation has frequently remained fragmented, with individual domains often taught separately rather than as an integrated approach to competency development (Johnson 2025a, 2025b).

The emergence of health promotion further broadened this perspective by redefining health as a dynamic process of enabling individuals and communities to improve control over their health rather than simply preventing or treating disease (World Health Organization 1986). Antonovsky's salutogenic theory complemented this view by proposing that health exists along a continuum and is strengthened through resources that promote comprehensibility, manageability, and meaningfulness (Antonovsky 1979, Mittelmark and Bauer 2017). Together, these complementary perspectives shifted educational priorities from disease-centered management toward adaptive functioning, resilience, participation, and person-centered care.

Recent conceptual developments have further strengthened this transition. Tatta *et al*. (2023) proposed the Pain Recovery and Integrative Systems Model (PRISM), which integrates salutogenesis, resilience, behavioral science, pain neuroscience, reflective learning, and lifestyle medicine into a recovery-oriented educational model. PRISM illustrates how pain education can intentionally cultivate adaptive capabilities alongside symptom management.

Similarly, Johnson (2025a, 2025b) expanded the biopsychosocial model by proposing an integral vision of pain grounded in a social model of health. This framework incorporates biological mechanisms, subjective experience, cultural meaning, environmental influences, community resources, and salutogenesis into a whole-person, whole-system perspective. By explicitly incorporating contextual and societal influences, Johnson reinforces the educational relevance of health promotion and adaptive recovery within contemporary pain care.

Concurrently, renewed interest in Saunders’ concept of Total Pain has reinforced the importance of multidimensional suffering beyond specialist palliative care. Contemporary authors recognize that chronic pain frequently reflects interacting physical, psychological, social, spiritual, and existential dimensions that cannot be fully explained by tissue pathology alone (Goebel *et al*. 2009, Habib *et al*. 2026). Rattner (2023) further argues that Total Pain should continue evolving conceptually to preserve its relevance across diverse chronic conditions while avoiding reductionist interpretations of human suffering.

Collectively, these developments demonstrate the progressive convergence of pain science, health promotion, and competency-oriented education. Although PRISM, Johnson's framework, and Total Pain provide complementary conceptual advances, each emphasizes different aspects of pain education and clinical practice. The present framework therefore seeks to synthesize these complementary educational contributions within a competency-based educational architecture that additionally incorporates Social Cognitive Theory, therapeutic alliance, physician professional sustainability, and longitudinal competency development. Rather than proposing a new conceptual model of pain, this framework aims to organize existing complementary theories into a coherent educational structure capable of informing curriculum design, educational implementation, and future empirical evaluation.

To clarify how the proposed framework extends rather than replaces existing conceptual models, Table 1 compares the principal contemporary frameworks informing pain science, health promotion, and pain medicine education, highlighting their major contributions, educational implications, current limitations, and their contribution to the proposed framework.

**Table 1:** Evolution of contemporary conceptual frameworks informing health-promoting pain medicine education and their contribution to the proposed integrated educational framework.

Framework	Primary focus	Main contribution	Educational implications	Current limitations	Contribution to the proposed framework
Engel (1977)	Biopsychosocial understanding	Biological, psychological and social integration	Person-centered thinking	Limited curricular operationalization	Conceptual foundation
Antonovsky (1979)	Salutogenesis	Adaptive health, Sense of Coherence	Health promotion and resilience	Not pain-specific	Health promotion foundation
Total Pain (Saunders; Goebel; Habib; Rattner)	Multidimensional suffering	Physical, psychological, social, spiritual dimensions	Holistic patient assessment	Originated in palliative care	Comprehensive understanding of suffering
PRISM (Tatta *et al*. 2023)	Pain recovery	Resilience, process-based education	Educational interventions for adaptive recovery	Physical therapy context	Educational resilience
Integral vision (Johnson 2025a, 2025b)	Social model of health	Whole-person, whole-system integration	Contextual understanding of pain	Not curriculum-oriented	Conceptual integration
Present framework	Competency-based pain medicine education	Integration of previous models	Longitudinal curriculum, competencies, assessment, physician sustainability	Requires empirical validation	Unified educational framework

## The proposed integrated health-promoting educational framework

The proposed framework does not replace existing conceptual models of pain but integrates their complementary strengths into a coherent competency-based educational framework for pain medicine. Building upon the conceptual foundations summarized in Table 1, it brings together six interdependent conceptual domains that collectively promote adaptive recovery, patient empowerment, professional sustainability, and health promotion (Fig. 1).

The framework is organized around six complementary domains, each fulfilling a distinct educational function while remaining dynamically interconnected. Rather than representing competing theories, these domains address complementary dimensions of competency development and person-centered pain care.

Total Pain provides the multidimensional understanding of suffering by recognizing that persistent pain extends beyond biological mechanisms to include psychological, social, spiritual, and existential dimensions (Saunders 1963, Goebel *et al*. 2009, Habib *et al*. 2026). This perspective encourages comprehensive assessment and person-centered care.

Salutogenesis and Sense of Coherence provide the health promotion foundation of the framework. Rather than focusing exclusively on pathology, they emphasize patients’ capacity to mobilize personal and environmental resources that enhance comprehensibility, manageability, and meaningfulness despite persistent pain (Antonovsky 1979, Mittelmark and Bauer 2017). Consequently, educational priorities shift from symptom control toward adaptive recovery, participation, and quality of life.

Resilience science contributes the dynamic processes through which individuals adapt to adversity. Contemporary literature increasingly conceptualizes resilience as a dynamic and modifiable capability associated with improved coping, psychological flexibility, self-efficacy, and functional recovery (Tatta *et al*. 2023). Accordingly, resilience becomes an explicit educational objective throughout professional training.

Social Cognitive Theory provides the behavioral mechanisms underlying competency development. Bandura's concepts of observational learning, self-efficacy, reciprocal determinism, and self-regulation support reflective learning, feedback, simulation, and behavioral change, while reinforcing patient self-management and shared decision-making (Bandura 1986).

The therapeutic alliance represents the relational dimension of the framework. Collaborative relationships based on empathy, trust, communication, and shared goals have consistently been associated with improved engagement, treatment adherence, and clinical outcomes (Flückiger *et al*. 2018). Consequently, relationship-centered care is regarded as a core educational competency rather than solely an interpersonal attribute.

Physician professional sustainability recognizes that effective pain care depends on clinicians who maintain resilience, reflective practice, psychological well-being, and professional meaning throughout their careers. Sustainable professional development therefore becomes both an educational objective and a prerequisite for compassionate, high-quality pain care.

Rather than functioning independently, these complementary domains interact throughout the educational continuum and are operationalized through a longitudinal competency-based curriculum supported by learner-centered educational strategies, authentic workplace-based assessment, and patient partnership (Fig. 1). The relationships illustrated in Fig. 1 represent conceptual educational pathways intended to generate hypotheses for curriculum development and implementation research rather than empirically validated causal pathways.

The educational value of the framework depends on how these domains are translated into curriculum design, teaching strategies, and competency assessment. The following section describes this educational implementation.

## Longitudinal competency-based pain medicine curriculum

The proposed framework extends beyond conceptual integration by providing a practical structure for competency-based pain medicine education. Rather than teaching the six conceptual domains as isolated topics, it advocates their progressive integration throughout undergraduate medical education, residency, fellowship, and continuing professional development. This longitudinal approach enables learners to progressively acquire, consolidate, and apply competencies related to multidimensional pain assessment, adaptive recovery, therapeutic alliance, patient empowerment, physician professional sustainability, and health promotion.

Implementation of the framework should align conceptual foundations with competency development through learner-centered teaching strategies, workplace-based learning, interprofessional education, and reflective practice. Across successive stages of training, the six domains should be revisited with progressively increasing complexity, clinical responsibility, and learner autonomy, allowing competencies to mature throughout the educational continuum.

Table 2 illustrates how each conceptual domain can be translated into educational objectives, teaching strategies, competency assessment, and expected health-promotion outcomes. Together, these components provide an educational blueprint that is sufficiently structured to guide curriculum development while remaining flexible enough for adaptation across diverse educational systems and healthcare contexts.

**Table 2:** Educational translation of the proposed integrated health-promoting framework for competency-based pain medicine education.

Framework pillar	Educational objectives	Learning strategies	Competency assessment	Expected health promotion outcomes
Total pain	Recognize the multidimensional nature of suffering and perform comprehensive biopsychosocial-spiritual assessment.	Patient narratives; case-based discussions; reflective practice; interdisciplinary teaching.	OSCE; workplace-based assessment; reflective portfolio.	Holistic assessment; person-centered care; improved patient understanding.
Salutogenesis & Sense of Coherence	Promote comprehensibility, manageability, and meaningfulness in chronic pain management.	Shared decision-making exercises; motivational interviewing; health literacy activities.	Reflective portfolio; Mini-CEX; structured feedback.	Patient empowerment; adaptive recovery; self-management.
Resilience Science	Develop adaptive coping, psychological flexibility, and recovery-oriented clinical reasoning.	Simulation; resilience workshops; mentoring; reflective writing.	Portfolio; multisource feedback; self-reflection tools.	Improved adaptation; reduced disability; enhanced participation.
Social Cognitive Theory	Strengthen self-efficacy, observational learning, and behavior change among learners and patients.	Role modeling; supervised clinical practice; peer learning; feedback.	Workplace-based assessment; EPAs; faculty observation.	Sustainable behavioral change; lifelong learning; patient activation.
Therapeutic Alliance	Develop communication, empathy, trust, and collaborative decision-making.	Standardized patients; communication workshops; interdisciplinary simulation.	OSCE; patient feedback; Mini-CEX.	Strong therapeutic relationships; improved adherence; patient-centered care.
Physician Professional Sustainability	Promote reflective practice, professional identity formation, resilience, and clinician well-being.	Mentoring; Balint groups; reflective portfolio; leadership development.	Reflective portfolio; multisource feedback; professional development plan.	Physician well-being; sustainable practice; resilient healthcare workforce.

## Patient partnership and educational implementation

Importantly, the framework recognizes patients as active educational partners rather than passive recipients of care Towle *et al*. (2010). Individuals living with chronic pain can contribute to curriculum design, patient narratives, simulation activities, reflective learning, communication training, and feedback on shared decision-making. Incorporating patients’ lived experiences strengthens comprehensibility, meaningfulness, empathy, therapeutic alliance, and person-centered care while fostering learner understanding of health promotion, patient empowerment, and collaborative practice.

As summarized in Table 2, the framework translates conceptual principles into observable educational competencies that support the development of physicians capable of delivering adaptive, equitable, and relationship-centered pain care throughout their professional careers.

Successful implementation, however, depends not only on curriculum design but also on assessment systems capable of evaluating adaptive, relational, and professional competencies alongside biomedical knowledge. This alignment between educational objectives, teaching strategies, and assessment is essential for competency-based medical education.

Implementation may encounter cultural and institutional barriers, particularly where pain medicine remains predominantly biomedical or procedure-oriented. Addressing these challenges will require faculty development, interdisciplinary collaboration, leadership support, and gradual integration of salutogenic principles into existing curricula rather than replacement of established educational models. Embedding these competencies within established competency-based medical education frameworks may facilitate institutional acceptance while promoting a progressive transition toward person-centered, health-promoting pain education.

## Educational assessment

Competency-based pain medicine education requires assessment that extends beyond biomedical knowledge to include adaptive, relational, and professional competencies. Accordingly, evaluation should combine traditional knowledge testing with authentic workplace-based assessment, direct observation, reflective practice, and longitudinal programmatic assessment.

Assessment of clinical competence should primarily focus on observable professional behaviors through educational tools such as Objective Structured Clinical Examinations (OSCEs), Mini-Clinical Evaluation Exercises (Mini-CEX), Entrustable Professional Activities (EPAs), workplace-based assessment, multisource feedback, patient feedback, and reflective portfolios. These methods evaluate competencies including communication, shared decision-making, clinical reasoning, professionalism, relationship-centered care, and person-centered pain management.

Psychometric instruments may complement educational evaluation by measuring learner- or patient-related outcomes rather than clinical competence itself. Examples include the SOC-13 or SOC-29 for Sense of Coherence (Antonovsky 1993), the Connor–Davidson Resilience Scale for resilience (Connor and Davidson 2003), and validated measures of the therapeutic alliance (Flückiger *et al*. 2018). These instruments should therefore be interpreted as complementary outcome measures rather than direct assessments of competency.

Rather than relying on isolated examinations, the proposed framework advocates programmatic assessment, integrating multiple sources of evidence collected longitudinally to support learner development, feedback, and continuous professional growth.

## Health promotion, equity, and social determinants of health

Persistent pain is influenced by biological, psychological, social, cultural, environmental, and economic determinants. Socioeconomic disadvantage, occupational exposures, stigma, health literacy, and inequitable access to healthcare substantially affect both pain experiences and treatment outcomes (World Health Organization 1986, Marmot *et al*. 2008).

Accordingly, pain medicine education should prepare physicians to recognize and address these determinants through competencies in cultural humility, trauma-informed care, advocacy, health literacy, and shared decision-making. By integrating these principles, the proposed framework aligns pain medicine education with the Ottawa Charter for Health Promotion, positioning participation, empowerment, equity, and person-centered care as core educational outcomes. Salutogenic education should complement—not replace—structural interventions aimed at reducing inequities in healthcare access and addressing the social determinants of pain.

## Future directions

The proposed framework provides a conceptual foundation for educational innovation and requires empirical validation across diverse educational and healthcare settings. Future studies should evaluate curriculum implementation, faculty development, competency assessment, implementation strategies, and educational acceptability.

Longitudinal multicenter research is needed to determine whether integrating health promotion principles into pain medicine education improves learner competencies, therapeutic relationships, patient activation, functional recovery, physician well-being, and healthcare system performance. Future implementation studies should also examine feasibility, contextual adaptation, and implementation fidelity across different educational environments.

Future work should additionally explore adaptation of the framework across other health professions involved in interdisciplinary pain management and evaluate its contribution to interprofessional education and collaborative models of care.

## Conclusion

Pain medicine is evolving from predominantly disease-oriented models toward salutogenic, resilience-oriented, and health-promoting approaches that recognize persistent pain as a multidimensional health condition rather than solely a biomedical problem. Although recent conceptual advances have expanded understanding of persistent pain, their translation into competency-based medical education remains limited.

This perspective addresses this educational gap by integrating Total Pain, Salutogenesis and Sense of Coherence, resilience science, Social Cognitive Theory, therapeutic alliance, physician professional sustainability, and competency-based medical education within a coherent educational architecture. By aligning these complementary domains within a longitudinal competency-based curriculum, the proposed framework offers a practical foundation for educating physicians capable of promoting adaptive recovery, patient empowerment, equitable person-centered care, and professional sustainability.

Rather than positioning health promotion as an adjunct to pain management, the framework places it at the center of pain medicine education. As a conceptual framework, it should be viewed as a hypothesis-generating educational model intended to guide curriculum development, implementation research, and future empirical evaluation rather than as a validated educational intervention.

Future studies should evaluate its feasibility, educational effectiveness, implementation across diverse healthcare settings, and potential impact on learners, patients, and health systems. If empirically supported, the framework may contribute to strengthening the integration of contemporary pain science, health promotion, and competency-based medical education in future pain medicine curricula.

## Data Availability

No new empirical data were generated or analyzed in this study. All information discussed in this article is derived from previously published literature.
